# The Long‐Term Clinical Outcomes of Triple‐Frequency Ultrasound in Treatment of Cellulite

**DOI:** 10.1111/jocd.71292

**Published:** 2026-11-11

**Authors:** Irina Chervinskaya, Nadezhda Kuprina, Ilja Kruglikov

**Affiliations:** ^1^ Clinic DK Forma St. Petersburg Russian Federation; ^2^ Eleos Medical Center St. Petersburg Russian Federation; ^3^ Wellcomet GmbH Bruchsal Germany

**Keywords:** cellulite, clinical study, LDM, triple‐frequency ultrasound

## Abstract

**Objectives:**

In this non‐interventional case‐series follow‐up study, we investigated the long‐term outcomes of triple‐frequency LDM (TF‐LDM) ultrasound in treatment of cellulite.

**Methods:**

Fourteen Caucasian women with mild‐to‐severe gynoid lipodystrophy were included in this study. All participants underwent long‐term follow‐up (LTFU) assessments between 17.0 and 32.5 months after treatment, and the obtained results were compared with the corresponding values at baseline, on the day of the last treatment, and at the intermediate follow‐up (IFU). Cellulite severity at LTFU was assessed by eight independent clinicians using the five‐grade Clinician‐Reported Photonumeric Cellulite Severity Scale (CR‐PCSS). Participant satisfaction was evaluated using the 11‐point Global Aesthetic Improvement Scale (GAIS). Thirteen participants also underwent B‐mode ultrasonography and real‐time compression elastography to objectively assess treatment outcomes by determining the elastic scores of the dermis and skin‐associated adipose tissue (SAAT) using a five‐grade color scale.

**Results:**

The mean value of CR‐PCSS score for the 14 participants at LTFU was 1.65 ± 0.85, compared with 2.30 ± 0.90 at baseline, 1.25 ± 0.86 on the day of the last treatment, and 1.04 ± 0.92 at IFU, respectively. Treatment outcomes continued to improve after the final TF‐LDM treatment session, and the clinical benefits remained evident for at least 22 months compared with baseline. Elastic scores increased significantly from baseline to IFU in both the dermis and SAAT. In the dermis, a slight but statistically significant reduction in elastic scores was observed at LTFU compared with IFU, whereas no statistically significant difference between IFU and LTFU was detected in the SAAT. The mean participant satisfaction score at LTFU was 9.29 ± 0.83. No treatment‐related side effects were reported during the treatment course, at IFU, or at LTFU.

**Conclusions:**

TF‐LDM is an effective, well‐tolerated, and well‐accepted treatment for cellulite that provides significant long‐term clinical improvement without treatment‐related side effects.

## 1 Introduction

A dimpled lumpy skin appearance in the gluteofemoral body area named cellulite remains one of the most common aesthetic concerns among women. Over the past decades, numerous energy‐based treatment modalities have been proposed for its management. Although these approaches are based on different physical principles—including vacuum massage, various types of radiofrequency currents, light‐based therapies, and high‐intensity ultrasound—the temporal pattern of their clinical outcomes has been remarkably similar: an initial phase of visible improvement followed by a relatively rapid return to the pretreatment severity of cellulite within several months after treatment cessation. Such short‐term improvements have been disappointing for both clinicians and patients, gradually undermining confidence in the ability of existing treatment modalities to achieve durable clinical outcomes. This is one of the main reasons why none of these approaches has become the gold standard for the treatment of cellulite.

As discussed previously, the limited efficacy of existing treatment modalities is largely attributable to an incomplete understanding of the pathophysiology of cellulite and, consequently, to the selection of inappropriate therapeutic targets [1, 2]. Recently, we proposed a novel pathophysiological model linking the development of cellulite to the selective accumulation of endogenous lipopolysaccharides (LPS) within the gluteofemoral white adipose tissue (WAT) of women [1]. According to this model, LPS accumulation induces a state of chronic low‐grade inflammation in this fat depot, particularly in its superficial compartment, known as skin‐associated adipose tissue (SAAT), which represents the human counterpart of dermal white adipose tissue (dWAT) in rodents [3]. We further proposed that the translocation of LPS from the intestine into adipose tissue occurs recurrently in women during the T4 subphase of the menstrual cycle. This process is thought to result from a transient increase in intestinal permeability occurring concurrently with corresponding changes in endometrium [2, 4]. The physiological nature of this mechanism may explain why cellulite develops in more than 85% of women over the age of 20 years.

Chronic inflammation in SAAT influences not just the physiology of this special and highly dynamic fat depot but also the morphology of the dermal‐hypodermal junction (DHJ), allowing protrusions of SAAT into the dermis, which cause the visual appearance of cellulite [1]. According to this pathophysiology, the main targets in cellulite treatment must be the reduction of inflammation in SAAT and the reinforcement of the epidermal, dermal, and SAAT layers, which must lead to reduction of protrusions of SAAT into the dermis and diminish the projections of these protrusions to the skin surface [2]. Consequently, the optimal treatment method for cellulite should combine the antiinflammatory treatment effect on the SAAT with a skin tightening effect in the skin and SAAT, which is not a trivial combination. Indeed, most of the proposed energy‐based methods for cellulite are based on the application of large forces or high temperatures to the skin, which normally increase the inflammation in the affected area. One possibility to realize both effects in one treatment provides the Local Dynamic Micromassage (LDM) technology, which allows a quasi‐simultaneous application of several ultrasound waves with different frequencies and demonstrates both pronounced anti‐inflammatory and skin tightening effects on the tissue [2, 5].

Our recent retrospective pragmatic case‐series clinical study on 20 Caucasian females with mildto‐severe grades of cellulite, who received the monotherapy with triple‐frequency LDM (TF‐LDM) technology, demonstrated strong and remarkably stable treatment outcomes [2]. Subjective assessment of the patients using the five‐grade Clinician‐Reported Photonumeric Cellulite Severity Scale (CRPCSS) [6], at baseline (timepoint T1), on the day of the last treatment (T2), and during the last followup control (T3) revealed the average values of CR‐PCSS at T1‐T3 over all participants of 2.22 ± 0.82, 1.18 ± 0.77, and 0.84 ± 0.77, respectively. This resulted in a highly statistically significant skin improvement between T1 and T2, T1 and T3, and even between T2 and T3. These findings were objectively supported by application of real‐time compression elastography, which revealed significant reinforcement of the epidermis, dermis, SAAT, and apparent reinforcement of the superficial fascia at follow‐up controls as compared to their baseline values. Altogether, these results demonstrated that TFLDM waves produce a significant and long‐time improvement of the cellulite skin with its further progressive amendment after cessation of treatment. These subjective assessments provided by six independent clinicians (raters) and supported by objective results of sonoelastography were further underpinned by assessment of the patients' satisfaction with treatment results, which revealed a high level of contentedness of 8.95 ± 1.49 based on the 11‐grade Global Aesthetic Improvement Scale (GAIS) scale [2].

One limitation of the study [2] was a high variability of follow‐up controls ranging between 3 and 18 months (8.35 ± 4.44 months), which did not allow us to make any conclusion about the characteristic decay times of treatment outcomes. On the other hand, except for one patient with undifferentiated connective tissue dysplasia (UCTD) having a follow‐up control at 18 months after treatment, we did not observe any significant decay in treatment outcomes even during the follow‐up controls up to 17 months [2]. This effect was explained by a very strong reinforcement of the dermis and SAAT observed with sonoelastography in patients treated with TF‐LDM waves.

In this study, we extended the follow‐up controls for the patients investigated in study [2] to 1732.5 months to better assess the stability of treatment outcomes and eventually determine their characteristic decay times.

## 2 Methods

### 2.1 Participants

Twenty Caucasian females with mild‐to‐severe gynoid lipodystrophy aged 27–53 years who received cellulite monotherapy with TF‐LDM were included in a previous study [2]. Individuals who did not finish the treatment course, individuals with advanced‐stage lipoedema, and individuals who received cellulite treatment with any other treatment methods during the last year were excluded. None of the participants had received GLP‐1 receptor agonist (GLP‐1RA) therapy or undergone any other treatment that could have induced rapid weight loss. Participants' body weight was not specifically monitored before, during, or after the treatment course, including at follow‐up assessments. Our rationale for this approach was that cellulite affects more than 85% of women over the age of 20, thus presenting a physiological rather than pathological condition, and its appearance does not correlate with BMI [1]. From these participants, a sub‐cohort of 14 individuals was recruited for the late‐time follow‐up (LTFU) controls provided in this study (Table 1). Attrition of participants during longitudinal studies over several years (in this study it was up to 3 years from the baseline timepoint) is a typical phenomenon. In our case the participants # 1, 6, 10, 16, and 20 from study [2] were excluded from the current study since they were unobtainable for LTFUs. Patient # 2, having 18 months follow‐up control, was excluded since she had no intermediate follow‐up (IFU) assessment. No one patient was excluded from the current study because of any side effects or dissatisfaction caused by lack of treatment efficacy.

**TABLE 1:** Subjects and their treatment parameters.

Subject	Age, year	Sessions	Follow‐up at T3, months	Follow‐up at T4, months
3	41	10	17.0	32.5
4	39	10	17.0	26.0
5	28	10	11.0	26.5
7	36	10	9.5	23.5
8	37	10	10.5	26.5
9	33	10	10.0	24.0
11	48	10	4.5	23.0
12	52	10	6.0	19.5
13	48	10	6.5	22.5
14	39	10	5.5	17.5
15	52	10	6.0	23.0
17	35	10	4.0	17.0
18	36	10	4.5	18.0
19	45	10	5.0	19.5

Age of participants presented in Table 1 corresponds to their age at baseline; the numeration of participants corresponds to the numeration given in the previous study [2]. Participant #19 included in this evaluation demonstrated a high laxity of the skin at baseline and was diagnosed with undifferentiated connective tissue dysplasia (UCTD). No participants received any additional cellulite treatments between the end of the previous clinical study and the corresponding LTFU provided in this study. Written informed consents were obtained from all patients.

### 2.2 Study Design

This was a non‐interventional case‐series follow‐up study in which participants, previously treated in study [2], underwent additionally both subjective assessments using the 5‐point photonumeric CR‐PCSS scale and objective assessments using sonoelastography to evaluate post‐treatment changes during follow‐up examinations. All participants included in this study were previously treated 1–2 times per week for 10 sessions [2] with LDM Triple device operating with TF‐LDM waves of 1/3/10 MHz and 3/10/19 MHz (Wellcomet GmbH, Germany). As demonstrated in a preliminary pilot study conducted with the same LDM Triple device by Dr. Kim Sojung (Llas Clinic, Seoul, South Korea), this number of sessions was sufficient to provide stable improvement in cellulite appearance (personal communication). During the treatment, the whole affected gluteofemoral area and the thighs were treated uniformly with a standard program “Cellulite” on each side of the body. A standard ultrasound gel, without any additional pharmacological drugs, was used during treatment as the coupling medium.

### 2.3 Clinical Assessment

In the previous study [2], participants were evaluated photonumerically using the standardized photographs by six independent raters at three timepoints: baseline (T1), on the day of the last treatment (T2), and at follow‐up (T3). In the present study, the same participants were evaluated at LTFU (T4) by eight independent raters, five of whom had also participated in the previous study, thereby ensuring continuity while increasing the number of evaluators. These three additional raters were asked to evaluate the photographs obtained at T3 to allow a more direct comparison of the outcomes at T3 and T4. Accordingly, the number of independent raters at T3 increased to nine, and the CR‐PCSS scores for T3 were recalculated based on the assessments of all nine raters. This approach was adopted to reduce individual scoring bias and improve the reliability of the clinical endpoints. To differ between two follow‐up controls at T3 and T4, they will be further named IFU and LTFU, respectively.

Cellulite severity was assessed using the five‐point Clinician‐Reported Photonumeric Cellulite Severity Scale (CR‐PCSS) (0 = none, 1 = almost none, 2 = mild, 3 = moderate, 4 = severe), which was developed and validated for the evaluation of cellulite severity in the gluteofemoral region [6]. Standardized pre‐ and post‐treatment photographs of the affected gluteofemoral area at T1–T3 were obtained in a dedicated photography room to minimize the influence of external lighting conditions. The photographs at T4 were taken under the same light conditions and with the same camera as in [2], which allowed for the comparison of treatment outcomes. Patient satisfaction was evaluated using the 11‐point Global Aesthetic Improvement Scale (GAIS), scored from 0 to 10 (0 = dissatisfied; 10 = fully satisfied).

Whereas attrition of participants from longitudinal studies can generally lead to biased results, preliminary calculation of descriptive statistics for the initial cohort of 20 patients from [2] and reduced cohort of 14 patients included in this study demonstrated no statistically significant differences between CR‐PCSS assessments at timepoints T1–T3, which allowed us to exclude a potential bias.

Thirteen of fourteen patients included in this study received B‐mode ultrasonography and realtime compression elastography (sonoelastography) using the ElastoScan technique (linear array probe LA3‐16A operating with frequencies of 3.0–16.0 MHz) with ultrasound scanner model HS50 (Samsung Medison, Republic of Korea) provided by the same experienced sonographer as in the previous study [2] to avoid the bias. The 5‐grade coloration scale (1 = predominantly red, 2 = mixed yellow/red, 3 = mixed green/yellow, 4 = mixed blue/green, and 5 = predominantly blue) was applied for assessment of tissue stiffness (elastic scores). Such assessment was provided for 13 participants since participant # 8, who received the CR‐PCSS evaluation, had no sonoelastography at LTFU.

While the follow‐up controls after TF‐LDM treatment were carried out at the Clinic DK Forma, ultrasonographic and sonoelastographic investigations were performed at the Eleos Medical Center, which explains why the terms of the follow‐up controls for sonoelastography deviated from those for photonumeric assessment in the same patients.

### 2.4 Statistical Analysis

Statistical analysis was performed using statistic software RStudio ver. 2024.12.1 + 563 (Posit PBC, Boston, USA) by an independent statistician. Given the relatively small number of participants, we adopted a triangulation approach to strengthen the reliability and validity of conclusions. This methodological framework integrates complementary distribution‐free and model‐based statistical methods to evaluate both qualitative and quantitative data, providing convergent evidence through cross‐validation of the obtained results. By combining multiple analytical approaches, triangulation reduces the risk of bias and enhances the robustness of the findings despite the modest sample size.

Ordinal scales with small sample sizes were analyzed non‐parametrically. Data were calculated for each participant and for the whole cohort at four timepoints T1–T4 and expressed as the means ± standard deviations (SD). Differences were considered statistically significant at *p* < 0.05. Q–Q plots were used to assess the distribution of the CR‐PCSS assessments at each timepoint T1–T4. Since the individual scales for these parameters are ordinal and coarsely graded and the total cohort comprises only 14 patients, normality was neither expected nor testable with adequate power. Consequently, the Wilcoxon signed‐rank test and the Mann–Whitney *U* test were applied, as these procedures require no distributional assumptions and are robust to skewness and outliers.

The repeated ratings obtained for each participant in this study were not statistically independent. Analyzing them as independent observations would constitute pseudo‐replication and could substantially inflate the Type I error rate. To account for the hierarchical structure of the data, we fitted a linear mixed‐effects model with raters specified as a random effect. Agreement between the distribution‐free Friedman test, which evaluates the within‐subject time effect, and the linear mixed‐effects model provides convergent evidence for the robustness of the observed treatment effects.

Because of the longitudinal study design based on repeated measures of the same parameters during an extended period of several years, a restricted maximum likelihood method was applied to fit the linear random intercept mixed‐effects (LRIME) model to provide unbiased estimations of the variance components. The LRIME model in the form CR‐PCSS (*t*) = β_0_ + β_1_t was applied to investigate the CRPCSS score progression over time, *t*, at timepoints T1–T4 as well as to calculate the effective decay time of treatment outcomes in months.

Additionally, Bayes factors were calculated for the ES data. This analysis was performed because of the relatively small number of participants and the low variability of the baseline measurements. Under these conditions, conventional significance testing cannot reliably distinguish between a true absence of an effect and insufficient statistical power. Bayes factors were included as an exploratory supplementary analysis and were not interpreted as confirmatory primary evidence. To control the family‐wise error rate associated with multiple comparisons, *p* values were adjusted using the Holm–Bonferroni correction.

## 3 Results

### 3.1 CR‐PCSS Assessment

The description of 14 participants included in this study, their treatment, and follow‐up times are presented in Table 1. The age of the participants was 28–52 (40.64 ± 7.06) years at baseline; each of them received 10 treatments with TF‐LDM and had the LTFU ranging between 17 and 32.5 (22.79 ± 4.14) months after the last treatment compared to 8.35 ± 4.44 months as in study [2] (Table 1).

The mean CR‐PCSS values and their corresponding standard deviations for all 14 participants at timepoints T1–T4, as obtained by descriptive statistical analysis, are presented in Table 2. The CRPCSS values for the cohort of 14 participants at timepoints T1‐T4 were 2.30 ± 0.90, 1.25 ± 0.86, 1.04 ± 0.92, and 1.65 ± 0.85, respectively (Figure 1). This corresponds to the skin improvement of 1.05 ± 0.34 (*p* < 0.002) between T1 and T2, of 1.26 ± 0.34 (*p* < 0.002) between T1 and T3, and of 0.65 ± 0.27 (*p* < 0.002) between T1 and T4. There was a statistically significant worsening of treatment outcomes at T4 compared to T2 (−0.40 ± 0.33, *p* < 0.002) and to T3 (−0.61 ± 0.26, *p* < 0.002). Application of the non‐parametric signed‐rank Wilcoxon test to the whole cohort of 14 patients revealed a statistically significant improvement of the mean CR‐PCSS values assessed by all raters per patient between T1 and subsequent timepoints T2–T4 (Table 3). Application of the Holm‐Bonferroni correction confirmed the statistical significance of these results.

**TABLE 2:** Assessment of the cellulite severity at baseline (T1), on the day of the last treatment (T2), at IFU (T3), and at LTFU (T4).

Patient #	CR‐PCSS (T1)	CR‐PCSS (T2)	CR‐PCSS (T3)	CR‐PCSS (T4)
3	2.50 ± 0.55	0.50 ± 0.55	0.44 ± 0.53	1.50 ± 0.53
4	1.50 ± 0.55	1.00 ± 0.00	0.44 ± 0.73	1.12 ± 0.35
5	2.50 ± 0.55	1.17 ± 0.41	1.33 ± 0.50	2.12 ± 0.35
7	1.00 ± 0.00	0.33 ± 0.52	0.00 ± 0.00	0.38 ± 0.74
8	2.00 ± 0.00	1.00 ± 0.00	0.67 ± 0.50	1.50 ± 0.53
9	1.33 ± 0.52	0.33 ± 0.52	0.33 ± 0.50	1.25 ± 0.46
11	3.17 ± 0.41	2.00 ± 0.00	1.22 ± 0.44	2.00 ± 0.53
12	4.00 ± 0.00	3.00 ± 0.00	3.00 ± 0.00	3.12 ± 0.35
13	3.33 ± 0.52	2.33 ± 0.52	2.22 ± 0.44	2.62 ± 0.52
14	2.17 ± 0.41	1.17 ± 0.41	1.11 ± 0.60	1.50 ± 0.53
15	2.33 ± 0.52	1.33 ± 0.52	0.89 ± 0.78	1.62 ± 0.52
17	2.00 ± 0.00	1.00 ± 0.00	1.00 ± 0.00	1.38 ± 0.52
18	1.50 ± 0.55	0.50 ± 0.55	0.22 ± 0.44	0.88 ± 0.64
19	2.83 ± 0.41	1.83 ± 0.75	1.67 ± 0.50	2.12 ± 0.64

**FIGURE 1:**
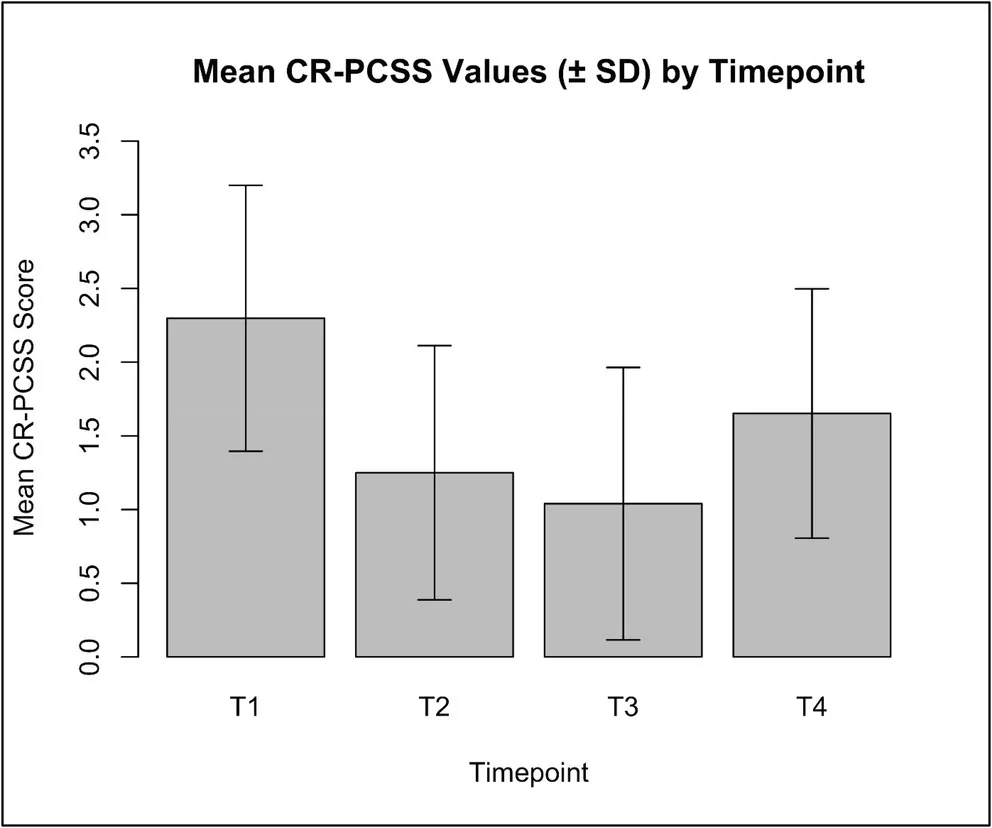
Estimated marginal means for CR‐PCSS values (±SD) by timepoints T1–T4 calculated with a LRIME model: 2.30 ± 0.90 (T1), 1.25 ± 0.86 (T2), 1.04 ± 0.92 (T3), and 1.65 ± 0.85 (T4). Compared to T1, the CR‐PCSS values at T2, T3 and T4 are significantly (*p* < 0.001) lower. This result was confirmed by application of the Holm–Bonferroni correction adjusting the *p* values to reduce the false positive errors.

**TABLE 3:** Mean CR‐PCSS differences between different timepoints after treatment.

Comparison	Number of pairs	Mean ± SD	*p* value
T1 vs. T2	14	1.048 ± 0.336	0.0010
T1 vs. T3	14	1.258 ± 0.344	0.0011
T1 vs. T4	14	0.646 ± 0.270	0.0011
T2 vs. T3	14	0.210 ± 0.258	0.0127
T2 vs. T4	14	−0.402 ± 0.332	0.0017
T3 vs. T4	14	−0.612 ± 0.261	0.0011

The average intraclass correlation coefficients for all raters, ICC (2,k), for the CR‐PCSS assessments at time points T1–T4 were 0.959, 0.952, 0.961, and 0.932, respectively, indicating high inter‐rater reliability.

Implementation of the LRIME model in the form CR‐PCSS (t) = β_0_ + β_1_t, where t is time in months after T2, to the whole cohort of CR‐PCSS data provided by all raters revealed the values *β*_0_ = 1.066 ± 0.220 and *β*_1_ = 0.021 ± 0.003 (*p* < 0.001). Model extrapolation beyond the maximum observed follow‐up time of 32.5 months estimated that, in the absence of additional TF‐LDM treatments after timepoint T2, the CR‐PCSS score would return to its baseline value of 2.30 after approximately 57.5 (95% CI: 36–87) months (Figure 2).

**FIGURE 2:**
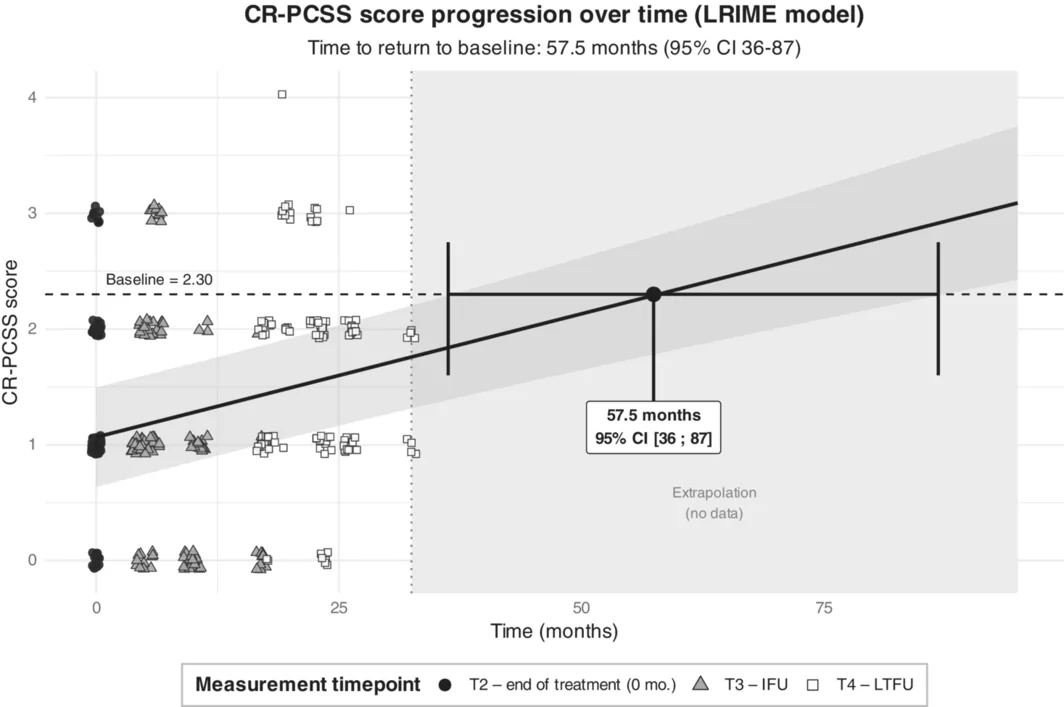
Application of a LRIME model with observation time in months to evaluate the CR‐PCSS score progression over time. The last observation time point was 32.5 months. Regression equation: CRPCSS (t) = β_0_ + β_1_t, β_0_ = 1.066 ± 0.220, β_1_ = 0.021 ± 0.003 (*p* < 0.001). Mean extrapolated estimated time to return to the average baseline CR‐PCSS score of 2.30 after TF‐LDM treatment is 57.5 (95% CI: 36–87) months.

### 3.2 Sonoelastographic Assessment

As we described in [2], ultrasonographic investigation of cellulite skin revealed a strongly irregular structure of the DHJ in patients with cellulite at baseline. The superficial fascia demarcating the boundary between the SAAT and the subcutaneous WAT appeared at baseline as a thin hyperechogenic layer. After treatment with TF‐LDM, the DHJ appeared more regular, and the superficial fascia appeared thicker than at baseline. Such improvements were also observed at LTFU controls provided 17–32.5 months after the last treatment, although they were less distinct compared to the results at IFU.

Assessment of elasticity of the epidermis/dermis and SAAT revealed significant reinforcement of these layers at both IFU and LTFU as compared to their baseline values (Table 4 and Figure 3). The average values for elastic scores obtained with sonoelastography at baseline and during IFU were 2.98 ± 0.57 versus 4.09 ± 0.53 (dermis) and 2.93 ± 0.65 versus 3.94 ± 0.63 (SAAT); at LTFU these values were 3.76 ± 0.59 (dermis) and 3.81 ± 0.57 (SAAT). Reliable ES‐assessment of the superficial fascia by SEG was not possible because of the thin thickness of this structure. Application of the Wilcoxon signed‐rank test demonstrated that the improvement in tissue stiffness at both IFU and LTFU was statistically significant in all investigated areas compared with baseline, with the exception of the epidermis/dermis layer in the right lateral area at LTFU (*p* = 0.08).

**TABLE 4:** Elastic scores (ES) of the dermis and SAAT at baseline and during the follow‐up sonographic investigation.

Area	SAAT, baseline	SAAT, IFU (T3)	SAAT, LTFU (T4)	Epidermis/Dermis, baseline	Epidermis/Dermis, IFU (T3)	Epidermis/Dermis, LTFU (T4)
Gluteal, l	3.07 ± 0.62	4.07 ± 0.62*	3.92 ± 0.67**	3.07 ± 0.47	4.28 ± 0.61*	4.00 ± 0.43**
Gluteal, r	3.00 ± 0.68	3.93 ± 0.62*	4.00 ± 0.60**	3.14 ± 0.36	4.14 ± 0.36*	3.75 ± 0.45***
Lateral, l	2.71 ± 0.61	3.86 ± 0.53*	3.50 ± 0.52***	2.86 ± 0.53	4.00 ± 0.39*	3.50 ± 0.67***
Lateral, r	2.86 ± 0.53	3.86 ± 0.53*	3.58 ± 0.51***	3.00 ± 0.55	4.07 ± 0.62*	3.58 ± 0.67
Femoral, l	2.86 ± 0.86	4.07 ± 0.62*	3.91 ± 0.51**	3.00 ± 0.78	4.07 ± 0.62*	4.00 ± 0.60**
Femoral, r	3.07 ± 0.62	3.86 ± 0.86**	3.92 ± 0.51**	2.86 ± 0.66	4.00 ± 0.55*	3.75 ± 0.62**

*Note:* Statistical differences between baseline and corresponding follow‐ups (Wilcoxon‐test).

* *p* < 0.001.

** *p* < 0.01.

*** *p* < 0.05.

**FIGURE 3:**
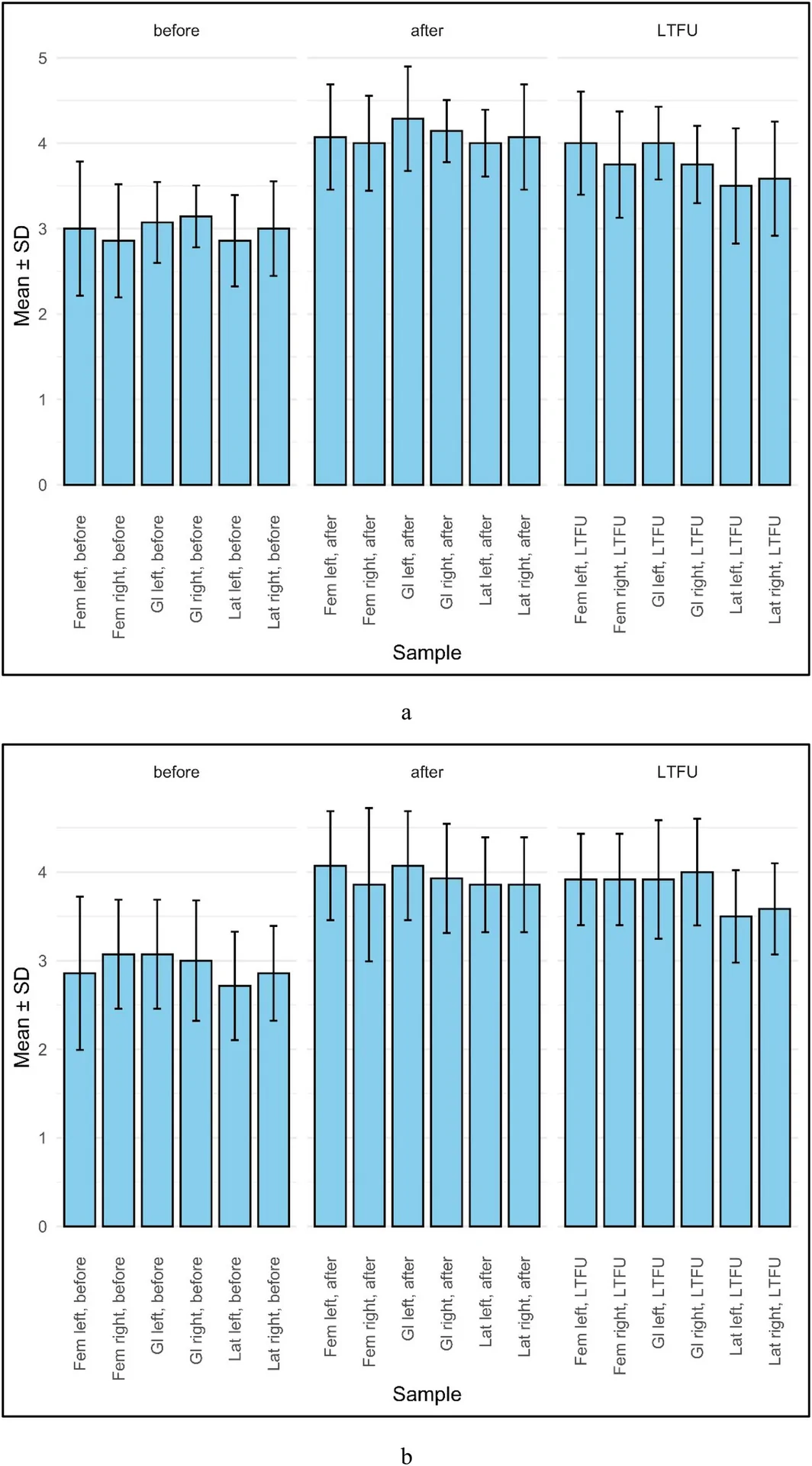
Elastic scores of the dermis and SAAT for six investigated areas (gluteal, lateral, and femoral) at baseline, IFU, and LTFU: (a) dermis, (b) SAAT.

Given the high statistical significance of most ES results presented in Table 4, we performed supplementary Bayesian *t*‐tests to further evaluate these findings, following the approach used in our previous study [2]. Bayes factors calculated for the paired baseline versus IFU and LTFU comparisons in the different regions of the dermis and SAAT confirmed the results obtained with Wilcoxon tests. These findings further support the conclusion that TF‐LDM treatment induced significant changes in the stiffness of both the dermis and the skin‐associated adipose tissue (SAAT) in most investigated areas, with these effects remaining evident not only at IFU but also at LTFU.

Application of the LRIME model revealed a significant main effect of timepoint on the elastic scores (Figure 4). In both the dermis and SAAT, elastic scores increased from baseline to IFU. In the dermis, a slight but statistically significant reduction in elastic scores was observed at LTFU compared with IFU. In contrast, no statistically significant difference in elastic scores between IFU and LTFU was observed in the SAAT (*p* = 0.17).

**FIGURE 4:**
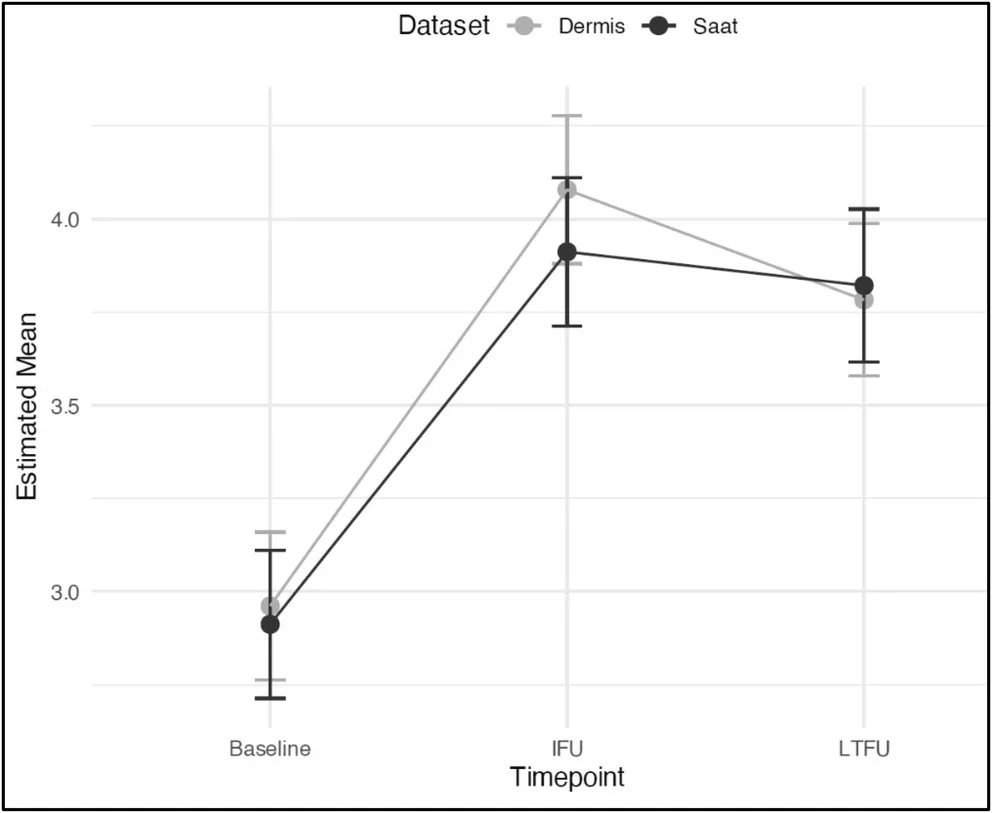
Estimated marginal means for elastic scores in dermis and SAAT calculated with a LRIME model.

### 3.3 Some Examples of Treatment Outcomes

Typical modifications of the cellulite skin and corresponding sonoelastographic images at baseline, after application of the TF‐LDM waves, at IFU, and at LTFU are shown in Figures 5 and 6. Figure 5 demonstrates the photographs of the patient #11 at baseline, after 10 treatments with TF‐LDM of 1/3/10 MHz (left side) and 3/10/19 MHz (right side), at 4.5 months IFU, and at 23 months LTFU for the relaxed and pinched gluteofemoral area (as assessed photonumerically). The average (assessed by all raters) CR‐PCSS score by this patient was improved from 3.17 ± 0.41 at T1 to 2.00 ± 0.00 at T2 (*p* < 0.05) and further to 1.22 ± 0.44 at 4.5 months IFU (T3) (*p* < 0.05). The CR‐PCSS score at 23.0 months LTFU was 2.00 ± 0.53; this score was significantly improved compared to its values at baseline (*p* < 0.001) but was worse compared to the CR‐PCSS score obtained at IFU. Figure 6 presents the sonoelastographic images for different parts of the gluteofemoral area of this patient. These findings clearly demonstrate that treatment with TF‐LDM significantly increases the stiffness of both the dermis and SAAT, and that this effect remains evident even 23 months after completion of treatment, at the LTFU sonoelastographic assessment.

**FIGURE 5:**
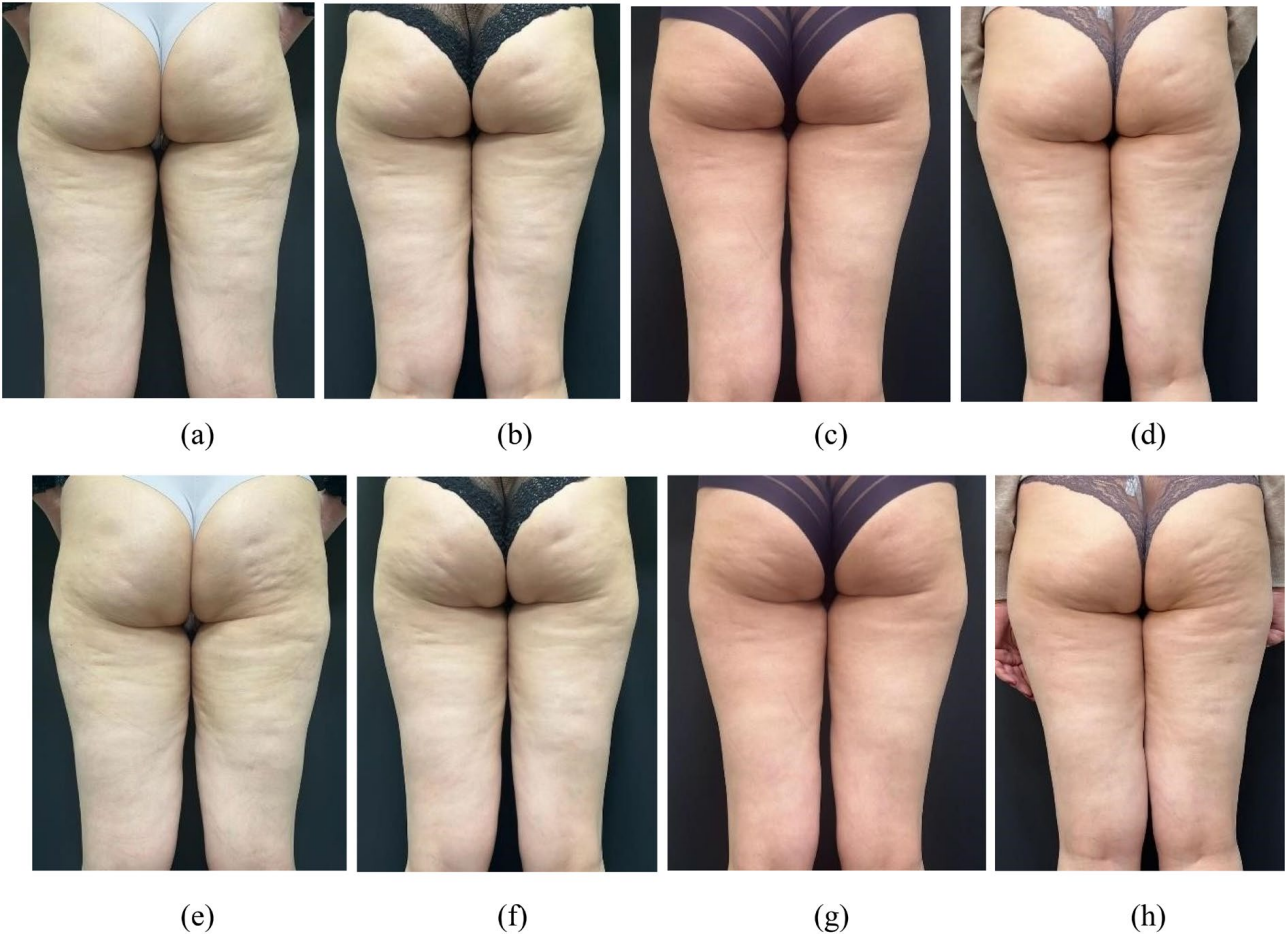
Patient #11: (a, e) at baseline, (b, f) after 10 treatments with TF‐LDM of 1/3/10 MHz (left side) and 3/10/19 MHz (right side), (c, g) at 4.5 months IFU; (d, h) at 23.0 months LTFU, (a–d) relaxed, (e–h) pinched.

**FIGURE 6:**
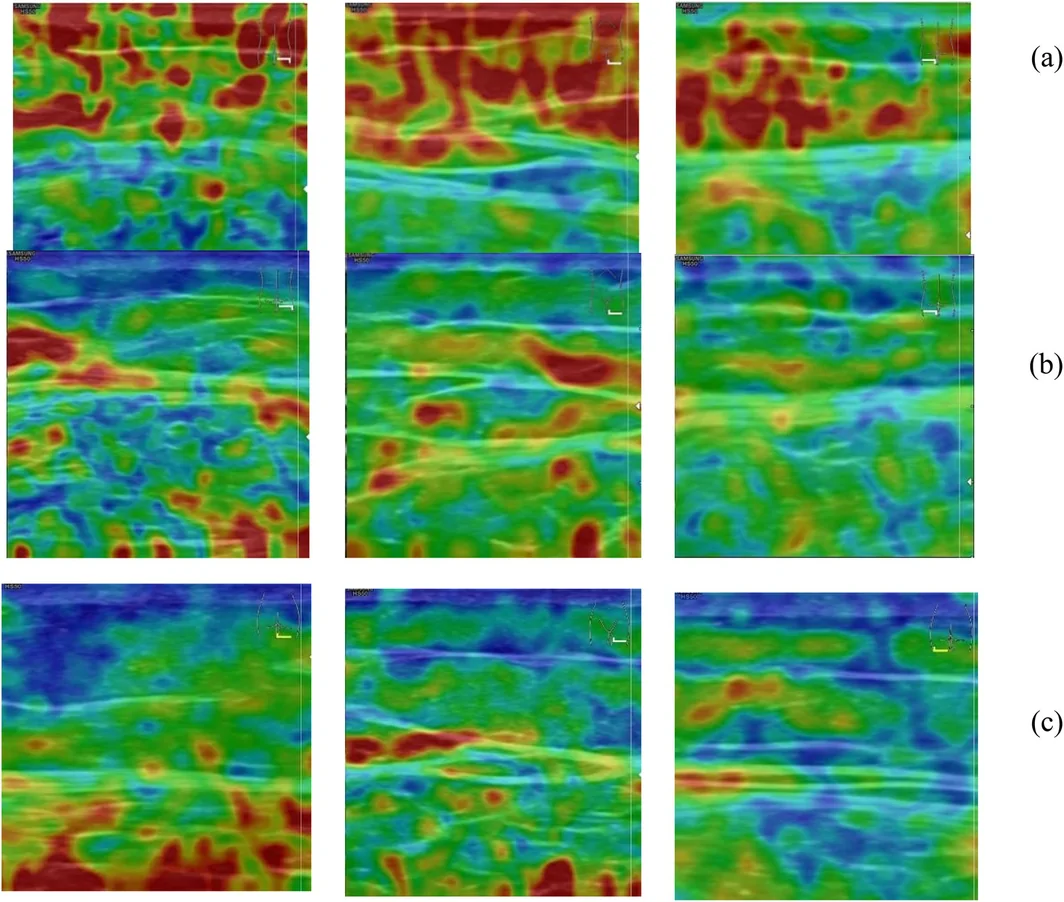
Sonoelastographic images of different parts of the gluteofemoral area (shown in the upper right of the images) of patient #11 (a) at baseline, (b) at 4.5 months IFU after 10 treatments with TFLDM of 1/3/10 MHz (left side) and 3/10/19 MHz (right side), and (c) at 16 months LTFU.

Additional examples comparing treatment outcomes at IFU and LTFU with corresponding baseline values are shown in Figures 7, 8, 9, 10. All these patients demonstrated a significant photonumeric improvement in cellulite severity (Table 2) accompanied by an increase in the stiffness of the soft tissues within the treated area following TF‐LDM therapy. At LTFU, both the clinical improvement and the increased tissue stiffness remained evident compared with baseline, although a slight reduction in CRPCSS treatment outcomes was observed between IFU and LTFU. For example, patient #19, who was diagnosed with UCTD, demonstrated the CR‐PCSS value of 2.83 ± 0.41 at T1 and of 1.67 ± 0.50 and 2.12 ± 0.64 at IFU and LTFU, correspondingly.

**FIGURE 7:**
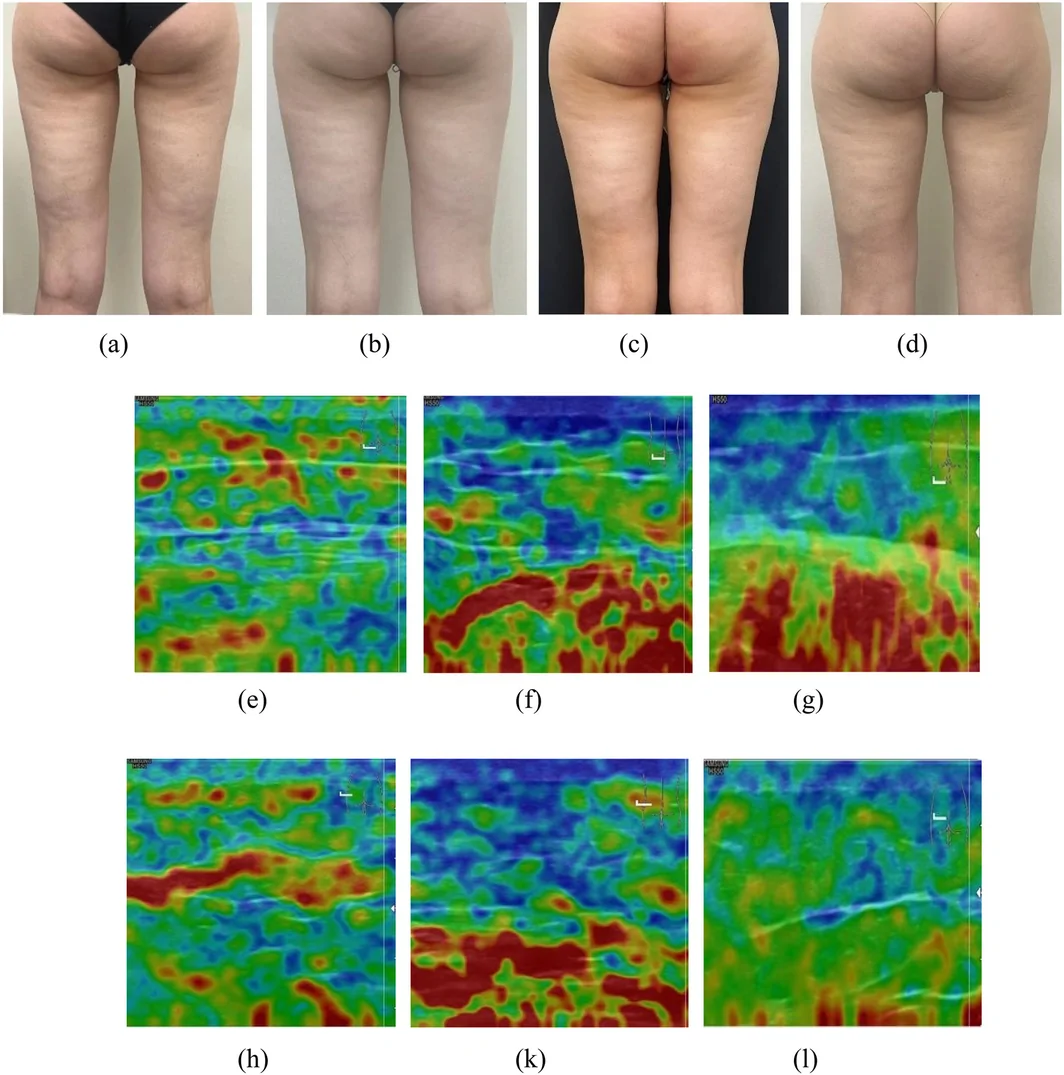
Patient #3: (a) at baseline, (b) after 10 treatments with TF‐LDM of 1/3/10 MHz, (c) at 17 months IFU, (d) at 32.5 months LTFU; (a‐d) relaxed. Sonoelastography of the left femoral (e–g) and left gluteal (h–l) areas: (e, h) at baseline, (f, k) at 7.0 months IFU, (g, l) at 32 months LTFU.

**FIGURE 8:**
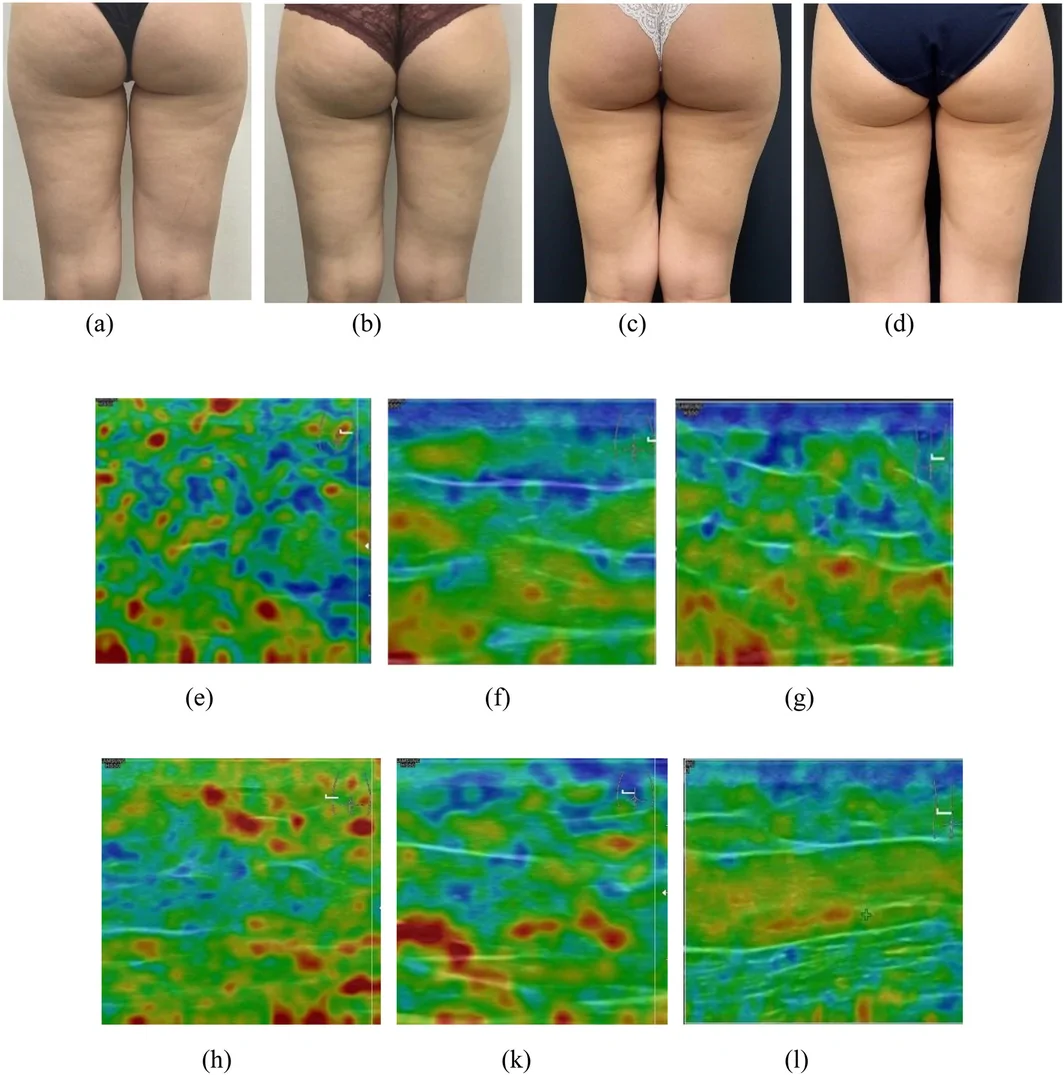
Patient #4: (a) at baseline, (b) after 10 treatments with TF‐LDM of 1/3/10 MHz, (c) at 17 months IFU, (d) at 26 months LTFU; (a–d) relaxed. Sonoelastography of the right (e–g) and left (h–l) gluteal area: (e, h) at baseline, (f, k) at 6.5 months IFU, (g, l) at 26 months LTFU.

No side effects were observed or reported by participants during the treatment with TF‐LDM, at IFUs or at LTFUs.

### 3.4 Satisfaction of Participants With Treatment Results

Assessment of the satisfaction of 20 participants with treatment results at IFU based on the 11‐grade GAIS scale in study [2] was 8.95 ± 1.49 (ranged between 5 and 10). Survey of the sub‐cohort of 14 participants in the current study revealed their satisfaction of 8.86 ± 1.70 (ranged between 5 and 10) at IFU and 9.29 ± 0.83 (ranged between 8 and 10) at LTFU. The differences between these assessments were not statistically significant. Notably, participants # 18 and 19 (Figures 9 and 10), who rated their satisfaction with treatment outcomes at IFU with 6 and 5 points, respectively, reported satisfaction scores of 10 and 9 at LTFU.

**FIGURE 9:**
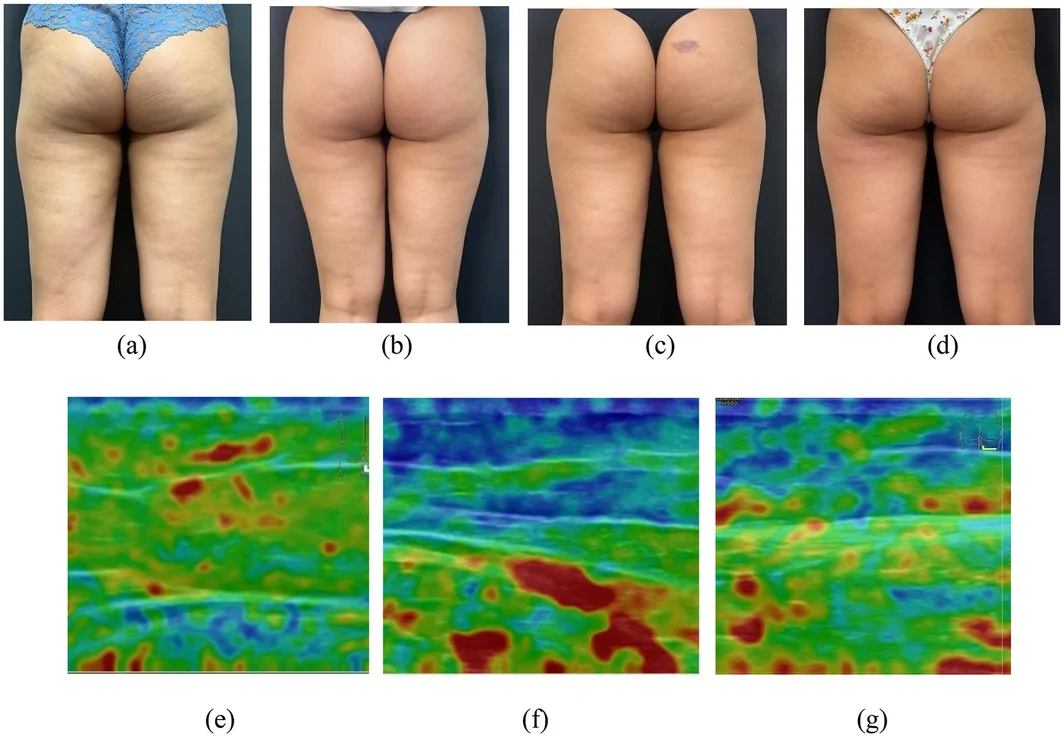
Patient #18: (a) at baseline, (b) after 10 treatments with TF‐LDM of 1/3/10 MHz, (c) at 2 months IFU, (d) at 18 months LTFU; (a–d) relaxed. Sonoelastography of the right femoral area (e–g): (e) at baseline, (f) at 2.0 months IFU, (g) at 19 months LTFU.

**FIGURE 10:**
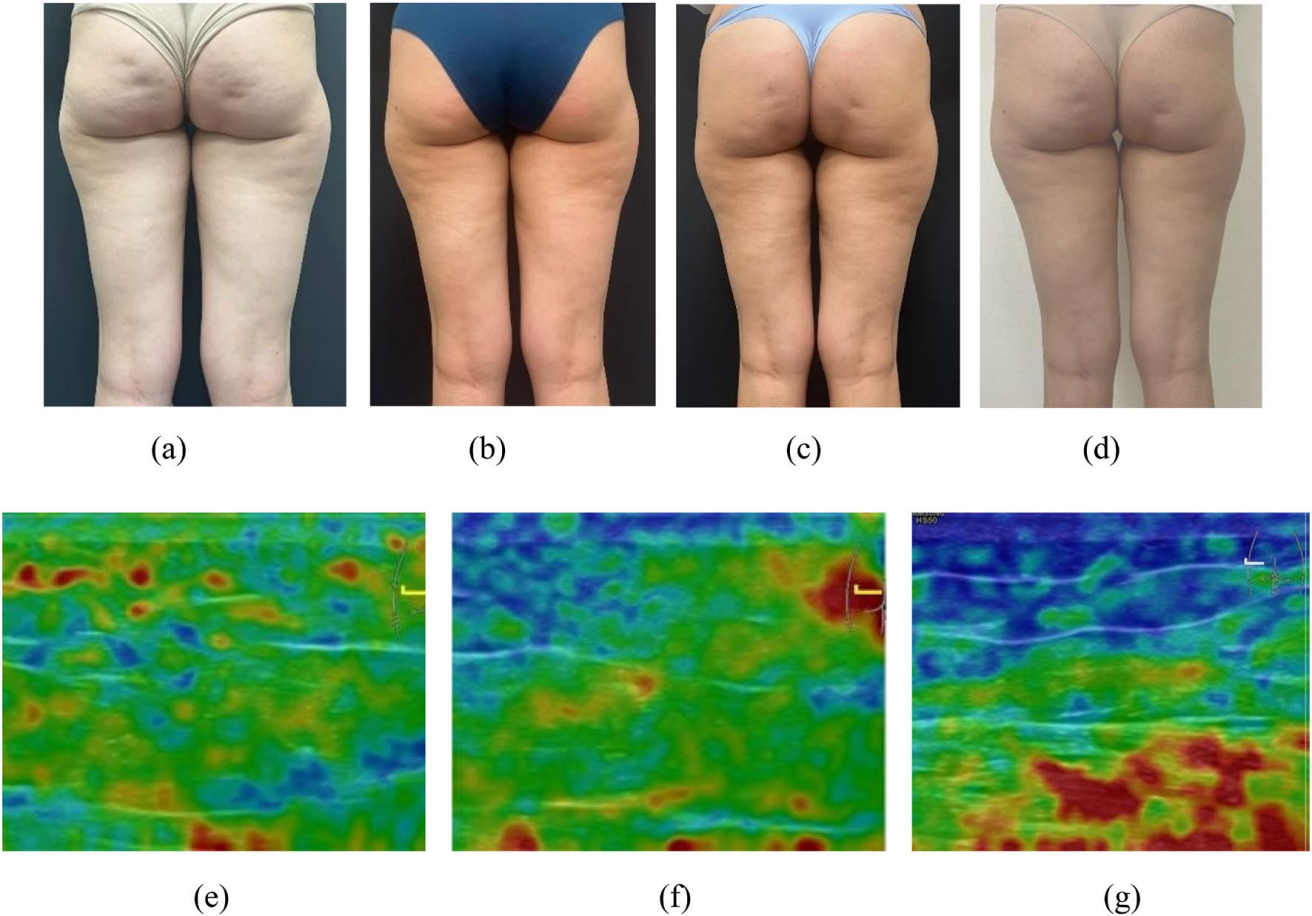
Patient #19 (with UCTD): (a) at baseline, (b) after 10 treatments with TF‐LDM of 1/3/10 MHz, (c) at 5 months IFU, (d) at 19.5 months LTFU; (a–d) relaxed. Sonoelastography of the left gluteal area (e–g): (e) at baseline, (f) at 4.0 months IFU, (g) at 20.0 months LTFU.

## 4 Discussion

The results of this study demonstrate a significant long‐term improvement in cellulite severity following treatment with TF‐LDM waves, as assessed at a mean long‐term follow‐up (LTFU) of 22.79 ± 4.14 months. As shown in Tables 2 and 3, the CR‐PCSS scores continued to improve between timepoints T2 and T3, with a mean interval of 8.35 ± 4.44 months for the entire study cohort. At LTFU (T4), all 14 participants exhibited higher CR‐PCSS scores than those recorded at IFU (T3), indicating a partial decline in the treatment effect (Table 2). Nevertheless, the CR‐PCSS scores for the cohort remained significantly improved compared with their corresponding baseline values (Table 3).

The mean improvement in cellulite severity for the entire cohort between baseline and LTFU was 0.65 ± 0.27 points on the CR‐PCSS scale (*p* < 0.002). These findings indicate that treatment outcomes continued to improve after the final TF‐LDM treatment session, and the beneficial effects of treatment remained evident for at least 22 months. Regression analysis using the LRIME model, in which the observation time (months) rather than the categorical timepoints (T1–T4) was included as the time variable, estimated an extrapolated mean time to return to the baseline CR‐PCSS score of 57.5 (95% CI: 36–87) months in the absence of additional TF‐LDM treatment after T2 (Figure 2). Although this estimate was derived from a linear random‐intercept model and may therefore overestimate the true duration of the treatment effect, it nevertheless supports the long‐lasting nature of the clinical benefits following TF‐LDM treatment.

The triangulation of the applied statistical methods further supported these conclusions. The extension of the statistical analysis from non‐parametric to model‐based approaches, together with the application of complementary statistical methods that are both sensitive and less sensitive to sample size, allowed a more comprehensive evaluation of the same data. The concordant conclusions obtained using the Friedman test and the linear mixed‐effects model provided convergent evidence for the robustness of the findings. The application of Bayesian analysis made it possible to distinguish between the true absence of an effect and insufficient statistical power. The calculated Bayes factors for the ES scores ranged from 32 to 100 or exceeded 100 in most of the investigated areas, indicating evidence ranging from *very strong* to *decisive* for the differences between baseline and follow‐up assessments. Overall, the statistical analyses demonstrated a high degree of convergence, with no complementarities or contradictions among the conclusions obtained using the different statistical methods.

As we proposed earlier [1], epidermis, dermis, DHJ, and SAAT must be sufficiently reinforced to reduce the protrusions of SAAT into the skin and thus diminish the appearance of cellulite. Reinforcement of the SAAT reduces its protrusions through the DHJ into the dermis, whereas reinforcement of epidermis/dermis reduces the residual projections of SAAT protrusions to the skin surface [2]. Sonoelastography revealed a significant increase in the stiffness of the epidermis, dermis, and SAAT at both IFU and LTFU in most of the investigated areas compared with baseline values (Table 4). This increase correlated with the improvement in the clinical appearance of cellulite, as assessed by the CR‐PCSS. Because the participants received no additional cellulite treatment after the final TF‐LDM session, it can be ruled out that the significant and sustained increase in tissue stiffness resulted from transient effects, such as edema. Instead, these findings suggest that the observed changes were associated with long‐term morphological modifications of the epidermis/dermis and SAAT.

The effective and long‐lasting reinforcement of the epidermis/dermis and the SAAT represents a remarkable property of TF‐LDM waves, as the mechanisms responsible for tissue reinforcement differ substantially between these layers. There remains considerable uncertainty in aesthetic medicine regarding the mechanisms underlying skin reinforcement following treatment with energy‐based devices. Traditionally, these mechanisms have been attributed to neocollagenesis (the formation of a new collagen within the skin), the development of fibrosis in the treated tissue, or collagen shrinkage, which is commonly proposed as the primary mechanism of thermal procedures. Although neocollagenesis can readily be demonstrated at the DNA, RNA, and procollagen levels, its contribution to the reinforcement of the mature collagen network, which primarily determines tissue stiffness, is generally limited. This is due to feedback mechanisms that activate matrix metalloproteinases, together with the inherently slow turnover of mature collagen in the skin [7]. The collagen shrinkage mechanism is well known from thermal capsulorrhaphy, where joint laxity is treated by thermal contraction of the joint capsule. However, the conditions under which collagen shrinkage occurs during capsulorrhaphy differ substantially from those encountered in non‐invasive aesthetic procedures [8]. Furthermore, it is well established that collagen denaturation (shrinkage) weakens rather than reinforces the extracellular matrix. Therefore, other mechanisms must be responsible for the reinforcement of the dermis observed in the present study.

Although the observed findings do not provide sufficient resolution to distinguish between the different microscale structural modifications occurring within the affected tissues, we propose here a theoretical mechanistic model describing the reinforcement effects of TF‐LDM waves on the epidermis/dermis and SAAT layers.

Sonoelastography estimates tissue stiffness by calculating the Young's modulus, defined as the ratio of uniaxial tensile stress to tensile strain (relative deformation), thereby characterizing the tissue's resistance to mechanical loading. Biological tissues are not purely elastic, but exhibit pronounced viscoelastic and, under certain conditions, plastic behavior, which is primarily determined by the extracellular matrix and the cytoskeletal architecture of their constituent cells. Consequently, the relationship between applied stress and the resulting strain is generally nonlinear. When soft biological tissue is subjected to mechanical loading, its stiffness changes in a time‐dependent manner, reflecting the balance between the storage and dissipation of elastic energy. Tissue stiffness generally increases under rapid loading and decreases when deformation is applied more slowly. Although several mechanisms contribute to this behavior, one of the most prominent is strain stiffening—the reinforcement of tissue resulting from load‐induced structural reorganization, which increases its resistance to further deformation and helps prevent irreversible mechanical damage. TF‐LDM therapy, which combines three ultrasound frequencies that alternate rapidly on the millisecond timescale, is capable of generating high deformation rates within soft tissues. Depending on the strain amplitude, which is determined by the applied ultrasound intensity, this mechanical loading may induce strain stiffening in the treated tissue. In addition, ultrasound has been shown to reinforce the cytoskeletal architecture of cells and to modulate intercellular junctions, thereby further contributing to increased tissue stiffness [9, 10].

Strain stiffening is a transient phenomenon with a characteristic decay time that depends on both the loading parameters and the mechanical properties of the affected tissue. Following a single mechanical loading event, the effect generally dissipates relatively rapidly. However, it is well established that fibrous matrices exhibit persistent mechanical responses after loading, resulting in the accumulation of residual stresses (mechanical memory) that enable the tissue to respond more effectively to subsequent loading cycles [11]. Consequently, achieving a stable structural modification of soft tissue requires not merely a pronounced response during a single treatment session but a course of repeated treatment sessions. The number of sessions required to induce a stable effect is expected to vary among individuals.

Three principal mechanisms can provide long‐term structural reinforcement of the dermis, all of which are closely associated with its three‐dimensional collagen network: (i) reorientation of collagen fibers in the direction of the applied uniaxial mechanical load, (ii) self‐reinforcement of the collagen network under cyclic compressive loading through the formation of new cohesive bonds between existing fibers, and (iii) increased collagen density through the synthesis of newly cross‐linked collagen fibers [12]. A spatially homogeneous (randomly oriented) three‐dimensional collagen network provides only limited mechanical reinforcement, reaching approximately 20% of the maximum stiffness achievable when the collagen fibers are aligned in the direction of the applied load. Reorientation of collagen fibers along the direction of mechanical loading can increase tissue stiffness several‐fold and should therefore be regarded as the primary response of the dermis to the quasi‐uniaxial mechanical loading generated by ultrasound waves [11, 12, 13].

Self‐reinforcement of the collagen network through the formation of new cohesive bonds between adjacent fibers represents another highly effective reinforcement mechanism. An increase in bond density of approximately 3% has been shown to increase material stiffness by approximately one order of magnitude [12, 14]. In contrast, reinforcement through the formation of an additional mature collagen network induced by mechanical loading should be regarded as a secondary mechanism. The mechanical contribution of newly synthesized collagen depends strongly on the so‐called length correction factor, which is determined by collagen fiber length, fiber diameter, interfiber spacing, and the interfacial bond strength between collagen fibers and the surrounding extracellular matrix [12]. Because this factor decreases rapidly as collagen fiber length decreases, this reinforcement mechanism is expected to be effective only when sufficiently long collagen fibers are present.

Furthermore, fibroblasts subjected to mechanical loading have been shown to generate contractile forces and to remodel the extracellular matrix by forming ligament‐like collagen straps that persist long after loading has ceased, thereby producing permanent plastic modification of the affected tissue [15, 16]. Such remodeling occurs when the applied mechanical loading exceeds the elastic limit of the tissue. Ultrasound waves are capable of both aligning collagen networks and promoting interactions between neighboring collagen fibers, thereby increasing their cohesion [17]. In the absence of standing waves within the tissue, both processes are expected to be mediated predominantly by ultrasound‐induced radiation pressure. In addition, TF‐LDM waves generate thermomechanical stress within the dermis and SAAT, producing localized mechanical deformation together with supraphysiological temperatures in these layers and substantial temperature gradients across the DHJ [18].

The mechanisms responsible for reinforcement of the SAAT differ fundamentally from those operating in the dermis [12]. WAT contains two distinct collagen networks: an intercellular network composed primarily of fibrillar collagens Types I and III, and a pericellular network consisting predominantly of collagens Types IV and VI. The mechanical properties of WAT are determined primarily by the pericellular rather than the intercellular collagen network [12, 19]. The Young's modulus of WAT increases linearly with the thickness of the pericellular fibrotic matrix and decreases with increasing mean adipocyte radius [19]. TF‐LDM waves significantly modulate expression of the mechanosensitive signaling protein caveolin‐1, which functionally interacts with transforming growth factor‐β (master‐regulator of collagen production) in the plasma membrane of adipocytes [20], and thus can substantially modulate the mechanical properties of SAAT. In addition, TF‐LDM exhibits pronounced anti‐inflammatory effects [5], which may reduce inflammation within the SAAT and thereby further limit its protrusion into the dermis. However, the precise contribution of these pathophysiological mechanisms to tissue reinforcement and stiffness remains to be elucidated and warrants further investigation in future studies.

While the present study was designed to address one of the main limitations of our previous study [2]—namely, the high variability and relatively short duration of the follow‐up period (3–18 months), which precluded conclusions regarding the characteristic decay time of the treatment effect—several limitations remain. Although the triangulation approach provided convergent evidence through crossvalidation of the obtained results and consistently supported positive treatment outcomes, the relatively small sample size may have limited the generalizability of the findings. Furthermore, because the original study [2] had a retrospective design, some degree of bias and incomplete demographic information cannot be excluded. As only Caucasian women were included, the findings cannot be generalized to other ethnic populations. While the occurrence of cellulite is not primarily determined by body weight or body composition, its clinical appearance and severity may be influenced by changes in these parameters. Consequently, changes in body weight, fat distribution, and muscle mass may, at least in some cases, affect the clinical appearance of cellulite. These variables may therefore represent potential confounders in longitudinal studies evaluating cellulite treatments [21, 22, 23]. However, they were not controlled for in the present study. Another limitation of this study is the confounding between rater panel composition and measurement time points. While the participants were evaluated by six raters at T1–T2, the rater pool changed to nine raters at T3 and eight raters at T4, as not all raters were available over a period exceeding 3 years. Consequently, the observed clinical worsening between T3 and T4 cannot be definitively distinguished from a potential rater effect. Although raters were included as a random effect in the statistical model, this approach cannot mathematically resolve the underlying confounding structure. Finally, although the proposed mechanism underlying the long‐term tissue reinforcement observed in this study is strongly supported by recent biophysical evidence, direct tissue‐level investigations will be required to validate the proposed mechanistic model. Additional prospective studies with larger patient cohorts will be needed to address these remaining limitations.

## 5 Conclusions

This clinical study demonstrated that treatment with TF‐LDM waves resulted in an improvement in cellulite severity, as assessed using the CR‐PCSS. The mean improvement from baseline was 1.05 ± 0.34 points at the end of treatment (*p* < 0.002), 1.26 ± 0.34 points at IFU (*p* < 0.002), and 0.65 ± 0.27 points at LTFU (*p* < 0.002). Thus, although the greatest improvement was observed at IFU, an improvement of 0.65 ± 0.27 CR‐PCSS points relative to baseline was maintained at LTFU (*p* < 0.002). This improvement was primarily associated with substantial reinforcement of the epidermis/dermis and skin‐associated adipose tissue. The mechanical modifications of these tissue layers were long‐lasting and remained evident for at least 22 months after completion of TF‐LDM treatment. TF‐LDM therapy was well tolerated, with no treatment‐related adverse events reported, and was well accepted by the participants, who reported a high level of satisfaction with the treatment outcomes.

## Funding

The authors have nothing to report.

## Ethics Statement

The present study was designed and conducted in accordance with ethical principles of the Declaration of Helsinki. The research was examined and permitted by the Ethics Review Board of the North‐West Public Health Research Center (protocol 2021/43.1 of 14.12.2021).

## Consent

All patients who participated in this study signed a written informed consent allowing to publish their data and images for scientific purposes.

## Conflicts of Interest

I.K. is the managing partner of Wellcomet GmbH. He was not involved in clinical treatment or assessment of the treatment results. Wellcomet GmbH provided support in the form of salaries for I.K. but did not have any additional role in the decision to publish or the preparation of this manuscript. The commercial affiliation of I.K. with Wellcomet GmbH did not alter the adherence to all journal policies on sharing data and materials. The other authors have no conflicts of interest to declare.

## Data Availability

The data that support the findings of this study are available on request from the corresponding author. The data are not publicly available due to privacy or ethical restrictions.
